# Noticeable Behavioral Differences Observed in Turkish Students Following Online Education

**DOI:** 10.3390/bs15040554

**Published:** 2025-04-19

**Authors:** Davut Hotaman

**Affiliations:** Faculty of Education, Davutpasa Campus, Yıldız Technical University, Gungoren, Istanbul 34165, Turkey; davut@yildiz.edu.tr

**Keywords:** behavior, COVID-19, education, online education, students

## Abstract

The COVID-19 pandemic, which posed a global threat, led many countries, including Turkey, to implement changes in their educational practices. In response to the “stay at home” directive aimed at preventing the spread of the virus, face-to-face education was suspended, and online education was adopted. As a result, children were unable to attend school for nearly two years. This sudden shift posed significant challenges for children, who were in the process of socialization and learning, as adapting to this new educational norm was not in alignment with their natural developmental needs. This study examines how staying at home affected the behaviors of children who were supposed to attend school, interact with their teachers and peers, socialize, and engage in learning. The research follows a qualitative phenomenological design, with the study group selected through criterion sampling. The collected data were analyzed using content analysis, leading to the identification of themes, categories, and codes. Particular attention was paid to participant and data saturation during the analysis process. The findings indicate that noticeable behavioral patterns were categorized under discipline, cognitive skills, social skills, motor skills, emotional skills, digital addiction, and personality traits across different educational levels. It is suggested that the type and frequency of these prominent behaviors observed in students may be associated with the shift to online education following the suspension of face-to-face learning due to COVID-19. Factors such as reduced peer interaction, diminished social engagement, and a lack of communication and interaction are considered to have played a role in these behavioral changes.

## 1. Introduction

Education is a broad field of activity that encompasses all forms of learning and teaching, whether intentional or incidental. Both formal and informal education are essential prerequisites for ensuring individual and societal development. The fundamental objective of education, whether delivered face-to-face or through online (internet-based) platforms, is to equip individuals—who are born with only a limited set of behavioral patterns—with socially and culturally desirable behaviors that are deemed necessary for life and approved by society (Demirel, 2021). Human beings inherently possess biological, psychological, and socio-cultural attributes (Bozkurt, 2006). From a socio-cultural perspective, an individual is a product of the society in which they are born and raised. Consequently, any differentiation in individuals can be traced back to the social and cultural influences of their immediate environment (Bilen, 2009; Brym, 1998). Society, in turn, constitutes an organic whole formed by the network of social relationships and interactions among individuals (Brym, 1998). Without society and its intricate web of relationships, the formation and development of individuals—both as social beings and as complete human entities—would be inconceivable.

### 1.1. Human Beings and Their Behavior

Human beings are bio-cultural and social entities (Bozkurt, 2006). While their biological traits emerge from the recombination of chromosomes inherited from their parents, their social and cultural attributes are shaped by their families and immediate surroundings. As both an influencing and influenced entity, human behavior is complex and multifaceted, often challenging to comprehend (Hotaman, 2018a). This complexity is attributable to the multitude of internal and external factors that govern an individual’s behaviors. As individuals interact with these factors, they adapt to changing circumstances and continue to navigate life. Therefore, in order to understand human beings, it is imperative to examine their behaviors and the underlying determinants of these behaviors. Human behavior, which is inherently intricate and multidimensional, is categorized into four primary domains: cognitive, affective, psychomotor, and social (Bacanlı, 2021; Senemoğlu, 2020). Cognitive behaviors, which are heavily reliant on mental processes and cannot be directly observed, include activities such as understanding, thinking, reasoning, perceiving, remembering, differentiating, and sequencing. These behaviors manifest indirectly through attention, perception, memory recall, inference-making, problem-solving, and imagination. Affective behaviors, on the other hand, pertain to emotional states such as interest, love, hatred, anxiety, and fear (Bacanlı, 2021; Senemoğlu, 2020). The third dimension of human behavior is psychomotor, or motor skills, which involve coordination between the mind and muscles. Actions such as walking, sitting, running, eating, drinking, jumping, assembling, and disassembling fall under this category (Bacanlı, 2021; Senemoğlu, 2020; Sönmez, 2009). Lastly, the social dimension of human behavior is linked to societal processes. Environmental stimuli play a crucial role in learning, as a child’s personality is shaped through the pairing of specific stimuli and responses from birth—a process known as contiguity (Özer & Hotaman, 2021).

### 1.2. COVID-19 and Online Education

COVID-19, also known as the novel coronavirus disease, first emerged in December in Wuhan, Hubei Province, China. Identified on 13 January 2020, the virus primarily affects the respiratory system and is characterized by symptoms such as fever, cough, and shortness of breath. The World Health Organization (WHO), considering the rapid increase in cases and the global response to the outbreak, declared COVID-19 a worldwide pandemic (Falowo, 2007; WHO, 2020). The classification of COVID-19 as a pandemic was largely due to its swift transmission across continents (Til, 2020). Although various measures were implemented to combat this highly contagious virus, social isolation policies—such as the “stay-at-home” directive—were particularly emphasized for individuals under the age of 20 and over the age of 65. Consequently, many countries discontinued face-to-face education at all levels and transitioned to internet-based *emergency remote education* (Yıldız & Bektaş, 2020). Despite being a technology-driven educational model, online education raises concerns about its capacity to deliver the full spectrum of learning experiences and skills offered by face-to-face instruction. While discussions surrounding online education primarily focus on educational objectives, the social, cultural, and psychological implications of enforced social isolation (particularly for young individual) have been largely overlooked under the shadow of a life-threatening pandemic. A review of the existing literature reveals several studies highlighting the deficiencies and challenges of distance education. Key issues identified include the lack of face-to-face interaction, which results in reduced engagement between students and instructors as well as among peers (Li et al., 2020; Moore & Kearsley, 2011); insufficient feedback mechanisms (Falowo, 2007); and inadequate support for both educational and professional development (Falowo, 2007) Additionally, the scarcity of student support services (Falowo, 2007) further exacerbated the challenges of online learning. The absence of instructional materials designed specifically for distance education, the failure of online education to replicate the social environment of schools, and the resulting lack of a sense of belonging were also reported (Falowo, 2007). Furthermore, the lack of experience among educators in delivering online instruction, coupled with curricula that were not adapted for remote education, reinforced the notion that online education fails to provide the same learning outcomes as face-to-face instruction (Li et al., 2020; Moore & Kearsley, 2011).

### 1.3. The School as an Educational and Social Environment

Beyond being a formal educational institution, the school serves as a crucial social institution that fosters an individual’s social, cultural, and psychological development. The educational process benefits both individuals and society, as it not only imparts knowledge and skills but also instills societal values, norms, and beliefs, thereby facilitating social integration. In schools, students articulate their common aspirations, projects, and expectations for life, engaging in discussions with their peers and learning how to coexist within a shared society (Gutek, 2006; Schlechty, 2006; Hotaman, 2018b). However, the shift to online education necessitated by the COVID-19 pandemic exposed numerous issues, including disparities in access to technology-based learning, disruptions to school and examination schedules, the inability to conduct teacher training sessions, challenges in delivering practical courses, economic disparities among families, the additional burden placed on parents and caregivers due to home-based learning, and the reduction of social interactions among children and adolescents, which led to social and physical isolation. Far from bridging the gap left by face-to-face education, online education struggled with its own inherent limitations. The pandemic highlighted that schools are not merely spaces for learning but also function as social environments that facilitate interaction, socialization, care, and guidance. Additionally, online education shed light on existing educational inequalities while underscoring the challenges of teaching and the broader roles that schools play beyond instruction (Anderson, 2020).

### 1.4. Present Study

Following a certain degree of success in combating the COVID-19 virus, face-to-face education was swiftly reinstated in Turkey, as in many other countries. However, upon returning to in-person learning, it was observed that students exhibited certain behavioral patterns that were not commonly seen before. From a developmental perspective, nearly all students had transitioned to the next stage of their development (e.g., from early childhood to middle childhood, or from preadolescence to adolescence). This shift, despite the resumption of face-to-face education, began to negatively impact classroom instruction and the teaching of subjects. Consequently, a research study was planned to investigate these behavioral changes by incorporating the perspectives of teachers actively working in the field. This study is significant as it examines the behaviors of students who, after nearly two years of online education, have returned to in-person learning, relying on the experiences and observations of teachers within school and classroom settings. The primary objective of this research is to analyze the behavioral changes observed in students across all educational levels—from preschool to high school—during the period of online education implemented as a substitute for face-to-face instruction due to the COVID-19 pandemic. This study aims to present its findings to parents, educators, and policymakers within the education system, ensuring that these behavioral changes are recognized and addressed appropriately. To achieve this objective, this study sought to answer two overarching questions: (1) “As a teacher, what were your experiences in the school and classroom upon returning to in-person education after COVID-19?” and (2) “What notable behaviors did you observe in your students following the period of online education implemented due to COVID-19?”

## 2. Conceptual Framework

The framework of this article revolves around examining the impact of the COVID-19 pandemic on children’s behaviors, particularly in the context of the transition to online education and prolonged stay-at-home measures. By synthesizing insights from the literature on human behavior, educational psychology, and the effects of social isolation, this research aims to provide valuable considerations for educators and policymakers worldwide, particularly in emerging economies, navigating the challenges of remote learning and addressing the social and developmental consequences of the pandemic-induced transition to online education.

## 3. Methodology

### 3.1. Research Pattern

In this study, phenomenology, one of the qualitative research designs, was employed. Qualitative research is a scientific method shaped around a central phenomenon that researchers seek to explore (Creswell, 2017; Merriam & Grenier, 2019; Merriam & Tisdell, 2015). The qualitative research process, which aims to understand and explain human experiences through a humanistic and interpretative approach, is influenced by various factors, including the nature of the social world and knowledge, the objectives of the research, the characteristics of the participants, and the researcher’s position (Merriam & Tisdell, 2015; Wilson, 2015). Therefore, qualitative research is a natural process that requires deep and prolonged interaction with individuals to explore their perceptions of the social world they inhabit (Wilson, 2015). Phenomenology, as a qualitative research design, seeks to uncover and define shared meanings derived from the lived experiences of multiple individuals regarding a particular phenomenon or concept (Creswell, 2017; Merriam & Grenier, 2019). The specific phenomenological approach adopted in this study is Heidegger’s hermeneutic interpretive phenomenology. This type of phenomenology involves both the description and interpretation of the phenomenon under investigation (Creswell, 2017; Merriam & Tisdell, 2015; Patton, 2018). Its primary focus is to reveal the details within experiences, considering their historical, cultural, and social contexts. Researchers employing this approach interpret the diverse meanings derived from participants’ shared experiences.

### 3.2. Study Group

In phenomenological qualitative research, selecting a study group involves reaching an adequate number of individuals who have fully experienced the phenomenon under investigation. Since there is no universal agreement in the literature regarding the “sufficient number of participants”, this study follows the recommendations of Creswell (2017) and Patton (2018), who suggest a sample size ranging between 5 and 25 participants. Accordingly, a homogeneous participant pool was created from teachers at different educational levels who had experienced the phenomenon firsthand. A total of 22 to 25 participants were selected using criterion sampling, a purposive sampling technique.

The rationale for employing purposive sampling was to ensure the inclusion of individuals who had experienced the phenomenon most directly, thereby ensuring homogeneity within the research group. The selected teacher participants were expected to provide data saturation, ensuring that a sufficient range of perspectives was covered (Hennink & Kaiser, 2022; Weber, 2019).

Table 1 presents information on 96 teachers who were enrolled in a Non-Thesis Master’s in Education Program at Yildiz Technical University’s Faculty of Education during the 2022–2023 academic year. These teachers were employed in various districts of Istanbul and worked exclusively in public schools. Their teaching experience ranged from five (5) to thirty-five (35) years, and they had been teaching in the same schools before the COVID-19 pandemic.

Data collection took place during the spring–fall semesters of 2022–2023, with the study group consisting of the following:

- 22 preschool teachers;
- 25 primary school teachers;
- 25 middle school teachers;
- 23 high school teachers.

In phenomenological research, the sample size may range from 2 to 25 participants, and working with an unbiased group of participants with relevant lived experiences contributes to a more accurate understanding of the phenomenon (Creswell, 2017; Patton, 2018). In accordance with these points, except for preschool and primary school teachers, the rest of the participants in this study were subject teachers specializing in Turkish, Religious Culture and Ethics, Information Technologies, Foreign Languages, Mathematics, Science, Turkish Language and Literature, and History.

### 3.3. Data Collection Tool and Process

The data collection tool used demographic questions regarding the teachers’ personal information (gender, seniority/experience, whether they worked in groups before COVID-19, and what educational level they taught at), as well as two open-ended general questions aimed at understanding and uncovering the phenomenon. In phenomenological research, participants are generally asked two questions (Creswell, 2017). Similarly, in this study’s data collection tool, two open-ended questions were used, believed to deeply uncover the phenomenon: (1) “What did you experience when you returned to school after online education, both in school and in the classroom?” and (2) “What notable behaviors did you observe in your students after online education?” These questions were designed to explore the phenomenon thoroughly. A structured interview form containing the research questions was prepared and sent via email to volunteer participants who had previously agreed to take part, with a notice indicating that their responses were expected within one (1) month. Given the difficulty of finding volunteer participants and collecting data in qualitative research, teachers who were willing to participate voluntarily were also allowed to join this study. To ensure the sample was reflective of unbiased observations, additional participants were included. In this way, sample saturation was sought. The data for the study were collected via email. Participants were informed about the use and confidentiality of the data, and their “voluntary participation consents” were obtained.

### 3.4. Data Analysis and Coding Process

All responses from the participants were numbered and archived in separate files in a computer folder categorized by educational levels: preschool, primary school, secondary school, and high school. In this study, the phenomenological research design was employed, and the written responses representing the participants’ views were analyzed using the “content analysis” technique (Merriam & Tisdell, 2015; Miles & Huberman, 1994; Yıldırım & Şimşek, 2018). The goal of content analysis is to identify the concepts and relationships that explain the collected data (Creswell, 2017). The process of data creation and analysis was carried out simultaneously. In this way, the phenomena under study were organized to help the readers better understand them (Merriam & Tisdell, 2015; Miles & Huberman, 1994; Strauss & Corbin, 1990; Yıldırım & Şimşek, 2018). The written views of the participants, a key feature of phenomenology, ensured that no participant’s viewpoint was lost, and this was beneficial in preventing meaning loss through repeated readings during the analysis. The responses from participants were archived and numbered in order of receipt (e.g., p1, p2, p3). The findings obtained through the data collection tool were repeatedly read by the researcher for content analysis. As the teachers’ responses from each educational level were reviewed, certain themes, categories, and codes began to emerge. Under the theme of “Notable Student Behaviors”, they were transformed into codes and categories (Merriam & Tisdell, 2015; Miles & Huberman, 1994). The teachers’ views were analyzed and converted into codes and categories, and the process continued until no new categories or codes emerged under the theme “Notable Student Behaviors Observed After Online Education”. This ensured both “thematic” and “meaning” saturation (Wilson, 2015). During this process, participants were constantly engaged and informed about the emerging themes, categories, and codes. “Participant confirmations” were obtained again. Some example expressions from participant views reflecting the codes and categories were presented in the findings section of the relevant educational level, preserving the content as is.

### 3.5. Validity and Reliability

Guba and Lincoln (2005) have emphasized that in qualitative research, the concept of credibility (trustworthiness) should be prioritized over validity–reliability, and they have identified certain criteria for this purpose (Merriam, 2009). These criteria include credibility, dependability, confirmability, and transferability (Wilson, 2015). Creswell (2017) also argues that at least one or more of these criteria should be stated in qualitative research. In this study, to ensure reliability and validity, “trustworthiness” was achieved through “prolonged involvement” and “member checking” with the participants (Streubert & Carpenter, 2011; Wilson, 2015). Almost all of the teachers participating in this study were students of the researcher in their graduate programs, and behavioral differences in students returning to school were discussed almost every week, with mutual discussions about what these behaviors were and what could be done about them. Feedback was provided to participants regarding the emerging themes, categories, and codes, and participant confirmations were obtained for any potential deficiencies, overlooked points, or misunderstandings. According to Yin (2014), dependability is achieved through the diversification of data and the creation of a chain of evidence. For this, “theory triangulation” and “researcher triangulation” were utilized. For theory triangulation, relevant published studies (from the printed literature) were used to enhance internal validity (Streubert & Carpenter, 2011). For researcher triangulation, to ensure the reliability of the research (Guba & Lincoln, 2005; Creswell, 2017), the content analysis conducted for each educational level was calculated using the “agreement/cohesion percentage” formula of Miles and Huberman (1994) [Agreement/(G.B. + G.A.)] × 100. The coding results developed by two doctoral students for each level were compared. For preschool, an agreement of 71.1% was achieved, for primary school 78.3%, for secondary school 77.4%, and for high school 80.2%. The agreement percentages obtained for the codings conducted for each educational level are above the accepted 70% threshold for social sciences (Yıldırım & Şimşek, 2018; Miles & Huberman, 1994).

### 3.6. Research Ethics and Implementation Process

The protection of volunteer participants and their stated experiences in a study is always the responsibility of the researcher (Merriam & Tisdell, 2015; Merriam, 2009). The researcher ensured the protection of participants’ names and gender status by coding the teachers’ responses. All confidentiality principles regarding data protection were adhered to. Furthermore, the researcher applied to the university where they are employed to seek an opinion regarding any potential ethical violations in the interview questions used in this study. The related application was evaluated in the Social and Human Sciences Research Ethics Committee Meeting of Yildiz Technical University on 2 June 2022, Meeting No: 2022.06, and an “Ethical Permission Certificate” was obtained.

### 3.7. The Researcher’s Participant Role

The most important role and responsibility of a researcher using the phenomenological design is to understand and reveal the impact of the research question on the participants’ “lived experiences”. According to Creswell (2017), the purpose of a researcher is to consider all aspects of a study. From the planning of qualitative research to the analysis, interpretation, and evaluation of data, all stages must be meticulously reflected in the measures taken to preserve the balance of interaction and impartiality between the researcher and the researched (Wilson, 2015). This reflection is achieved through the researcher keeping continuous field notes about themselves and the participants. This strategy is used to ensure the rigor, confirmability, and impartiality of the study, as well as to observe data saturation (Hennink & Kaiser, 2022; Weber, 2019).

### 3.8. Research Process

This qualitative phenomenological research was conducted according to the following procedural steps: (1) The research problem, which had been noticed and felt, was identified. (2) Based on the belief that the most suitable design for the identified research problem was qualitative phenomenology, the qualitative phenomenological design was chosen. (3) The phenomenon of this research was defined. (4) As the researcher, I distanced myself from judgments related to the phenomenon and sought out individuals with experiences related to the phenomenon, who were then located. (5) In the later stages of this research, a rich pool of volunteers who had experienced the phenomenon and were likely to participate in this study was created. (6) From this pool, a sufficient number of participants who could clarify and deepen the research topic were selected based on the sampling method of this study. (7) The research questions to be directed to the participants in the phenomenological study were determined. (8) The questions were compiled into an interview form, which was then sent to the participants’ email addresses via a link, asking them to respond within one month. (9) While the process of answering the research questions was ongoing, continuous meetings were held with the teachers who volunteered to participate, ensuring the establishment of trust with them. (10) Responses received within the one-month period were numbered and grouped into categories of preschool, primary school, secondary school, and high school, and began to be organized digitally. (11) Once “participant saturation” was reached within the response time, the analysis of the responses from the participants obtained through the interview form began. (12) All participant responses were read repeatedly, sorted by educational level (preschool, primary school, secondary school, and high school). (13) After several readings, markings and notes were made using colored pens and paper. (14) This process continued until the views of each participant had been read repeatedly and certain “themes, categories, and codes” became repetitive, indicating that “data saturation” had been reached (Hennink & Kaiser, 2022; Weber, 2019). (15) The obtained themes, categories, and codes were sent to the participants for “member checking”. (16) The results of the content analysis conducted for each educational level were analyzed using a second set of codes developed by two doctoral students, and the “agreement percentages” were examined using the formula of Miles and Huberman. The resulting agreement percentages are provided under the section titled “Validity and Reliability”. (17) While writing the article for this research, some of the participants’ views that revealed the phenomenon were added to contribute to “structural and textual description”. (18) Finally, phenomenological research concludes with a descriptive section that thoroughly explains “what” and “how” experiences were lived, after which the essence of the individuals’ experiences is revealed, evaluated, and discussed. In this way, *the essence* of a phenomenological study was reached (Creswell, 2017).

## 4. Findings

In this study, noticeable behavioral differences observed in students after online education were examined according to teacher opinions. The data obtained were analyzed by content analysis and transformed into themes, categories, and codes and are presented below according to education level.

Below are some of the teacher opinions coded as a result of content analysis, as shown in Table 2:

*“They can’t follow the instructions, can’t solve their personal problems, their attention span seems to be reduced, they can’t communicate efficiently and effectively, they have difficulty in making friends, holding a pencil, drawing is very poor, self-care skills are insufficient, they are not interested in the lesson, they have a high interest in digital”*.

(p4)

*“They don’t like rules, there is a significant increase in behaviour problems. They struggle with line works. It seems that there is an increase in attention, perception and focus problems. They cannot solve their own problems. They are very impatient. While their interest in activities is low, their interest in digital media seems to have increased. They cannot separate from their mothers”.*

(p7)

*“They have difficulty in focusing their attention. Their interest in activities is at a low level. They have a high desire to see their mother and go home. They are inadequate in playing and communicating with their peers. They have an interest in visual stimuli. They get bored in classroom activities. They don’t want to talk and express their emotions. Their drawing skills are very basic and they can’t hold a pencil properly”.*

(p19)

*“There were general difficulties in socializing with peers. They exhibited behaviors such as not knowing how to share, being stubborn in every matter, and initiating but failing to sustain verbal communication. They simply did not know exactly what expressions to use when initiating and maintaining communication with their peers”*.

(p20)

Some of the teacher opinions coded in Table 3 are provided below as follows:

*“We had learning difficulties, especially with foreign students. Since we do reading and writing with online education, the layouts and writings of the notebooks are very distorted. There is difficulty in writing. They have difficulty adapting to school rules. There are problems making friends, fitting in, and communicating. Interest in the course is low. Anxiety and worry are felt”.*

(p2)

*“While I thought that I had achieved the gains in online education, I saw that the success decreased by half when the children returned to school. They can’t be controlled, we’re facing an insane audience”*.

(p3)

*“At the beginning of the year, I encountered a group with significantly underdeveloped language skills. Their proficiency in Turkish has declined, and they have incorporated abbreviations into their spoken language. Their verbal communication remains underdeveloped, as they do not construct proper sentences. There are evident regressions in language development, such as an inability to express themselves properly and a reluctance to speak”*.

(p7)

*“There is a lack of attention. Difficulty in following rules and norms. Their interest in the lesson is insufficient. They fell behind in literacy. Adult dependence is observed. They always want to spend time in front of the screen. Inability to reach the gains, learning difficulties. Angry behaviour is observed”*.

(p12)

Below are some of the teacher opinions coded in Table 4:

*“Difficulties in following school rules, anger control issues, aggression, low academic achievement, lack of focus and attention, difficulties in reading books and solving tests, insufficient peer communication skills, indifference towards classes, social media and computer addiction, and observation of violence and swearing”.*

(p1)

*“Behavioural problems are observed in most of the students. Nobody adheres to school rules and norms. In addition, there are regressions in fine motor skills in accordance with their age. They have difficulty in taking notes. They do not read. There are difficulties in making friends and selfishness is observed”*.

(p3)

*“There is a discipline problem, they have forgotten the rules. They are aggressive. Since they have never had exams in the fifth grade, they experience learning difficulties in the sixth grade. There is a problem with attention span. They do not know where and how to talk. They never let go of their phones. They are angry”*.

(p7)

*“I estimate that only about 30% of the intended learning outcomes and objectives were achieved in the first grade following online education. Online learning has been a disadvantage for some students, as those in the literacy development phase have had limited opportunities to recognize, perceive, and observe sounds in an online environment. Furthermore, students lacking internet access and technological infrastructure were unable to participate in online classes, hindering their ability to meet educational goals”.*

(p23)

Below are some of the teacher opinions coded in Table 5 as follows:

*“Students have behaviour problems. They have forgotten where and how to talk. They have forgotten the rules of the school and the classroom. There is aggression. Profanity and vulgar language are frequently observed. Screen addiction and social media seem more important than anything else. Their attention easily gets distracted. Learning deficiencies are observed. Communication problems are observed. Interest in the lessons is low”*.

(p3)

*“There are problems with adhering to class rules. Non-compliance with norms and use of profanity is common. Learning deficiencies and difficulties are observed. Communication problems are observed. Interest in the lessons is quite low, and aggressive behaviours are observed”.*

(p6)

*“Their attention is scattered, and they have difficulty focusing. Except for a few students, who seem to care about the lessons, the rest have learning deficiencies. Unfortunately, their interest in the lessons is very low. Social media is very popular among students. They request slides. Their personality development seems problematic. They are unable to be themselves, have forgotten to share, are introverted, closed to communication, and seem dull”*.

(p17)

*“Some learning objectives were not met during online education. Despite participating in lessons through a screen, it seems as if they learned nothing—almost as if their knowledge has vanished”*.

(p19)

## 5. Discussion and Conclusions

This study examines the notable behaviors frequently observed in school and classroom environments among students returning to in-person education after COVID-19, based on teacher observations. Content analysis of the research findings reveals that striking behaviors observed among preschool, primary school, middle school, and high school students were commonly categorized under “discipline, cognitive skills, social skills, emotional skills, digital addiction, and personality traits”. Additionally, among preschool, primary school, and middle school students, “motor skills” also emerged as a distinct category in which notable behaviors were coded.

When examining the research findings, it becomes evident that at the preschool education level, the most notable behavior coded under the discipline category was “difficulty in following rules”; and at primary, middle, and high school levels, this issue was observed as “difficulty in adhering to norms”. Additionally, at middle and high school levels, behaviors such as aggression and the use of slang/profane language were prominently experienced. As teachers frequently observed instances of indiscipline, they began to associate academic failures with these behavioral issues. Education is a “deliberate process of enculturation”, and discipline is fundamental to the teaching and learning process. The increased prevalence of such previously uncommon behaviors has been interpreted as a contributing factor to emerging educational challenges. Negative behaviors such as aggression and violence among students may be linked to deficiencies in social problem-solving skills. Moreover, these behaviors may stem from the fact that student groups had been physically separated for nearly two years due to the pandemic, leading to a renewed need for self-assertion and social dominance. Similarly, the use of slang and profanity can be considered a form of aggression (Yavuzer & Üre, 2010). It should also be noted that the anxiety caused by COVID-19 has affected individuals differently; some students may be more prone to anger and emotional instability, leading them to use profanity or offensive language as a means of expressing aggression.

Some of the noticeable behavioral differences observed in students are also categorized under cognitive skills. These, in preschool students, include difficulty in following instructions, problem-solving, and maintaining attention; in elementary school students, difficulty in learning and maintaining attention; in middle school students, difficulty in learning and maintaining attention; and in high school students, difficulty in maintaining attention and learning deficits. It is understood that preschool students have difficulty following instructions related to classroom activities, which may be linked to a relatively long period of face-to-face education hiatus. This could be especially challenging for young children who have not yet achieved sufficient cognitive development. It is further understood that students in this age group may struggle with meeting some of their personal needs, concentrating, and solving minor problems on their own. Moreover, not only in preschool students but also in primary, middle, and high school students, difficulty in maintaining attention has been observed. In their research based on parental opinions after the COVID-19 pandemic, Mutlu et al. (2020) reported that parents also observed attention deficit in their children. Additionally, the dynamic and powerful presence of social media is considered a potential distraction for children in this age group, which could contribute to cognitive obstacles, possibly reflecting their developmental stage. Moreover, online education, which takes place over the internet, is thought to potentially contribute to attention deficits and focus problems. Attention, which is related to cognitive processes, refers to the concentration of consciousness on specific tasks. It is clear that attention and perception play critical roles in the effective learning of key subjects. In the study by Bakırcı et al. (2023), attention deficits were observed in students, and it was noted that these students began to learn more slowly and exhibited academic deficiencies. Additionally, Wendel et al. (2020) found that symptoms of attention deficit and hyperactivity disorder (ADHD) increased in children during the COVID-19 pandemic, according to interviews with parents of 4–5-year-old children. The noticeable behavior of struggling with learning, coded under the cognitive category, may possibly emerge as a result of this. Research conducted in different countries during the COVID-19 pandemic reveals that online teaching activities have not reached the learning levels achieved through face-to-face education (Kuhfeld et al., 2020). Research suggests that long breaks and disruptions in education are associated with learning difficulties and potential learning losses (Baz, 2021; Kaffenberger, 2021; Kuhfeld et al., 2020). In their research conducted after COVID-19, Bakırcı et al. (2023) also found that students learn slowly and with difficulty, and they have academic learning deficiencies.

The research findings indicate that teachers observed noticeable student behaviors in the social skills category as well. It is understood that participating teachers noticed inadequate social skills, which are necessary for young individuals and should be acquired during the relevant social development period, in categories such as “struggling to make friends, communicate, and adjust with peers”. However, a person is a whole with their environment, and this wholeness can be achieved by making friends and establishing healthy relationships with them. When young individuals are unable to achieve this, their social, emotional, and personality development could be negatively affected. Social skills are important for individuals to establish a place for themselves within the social structure they belong to and to be a part of that structure. Socialization is the process of acquiring societal and social skills, such as “reflecting emotions, thoughts, and experiences through mutual exchange to integrate with the outside world, collaborating, and working toward common goals within a group” (Çubukçu & Gültekin, 2006; Özden, 2000). It can be said that friendship relationships are an important need that cannot always be met within the family and are a constant source of social support. According to Wentzel (2003), children try to form interpersonal relationships at school every day and maintain these relationships. It can be said that the ability to form healthy and satisfying human relationships is one of the most important variables determining individuals’ physical and psychological well-being (Yüksel-Şahin, 1997, 2020). According to Piaget’s theory of cognitive development, cognitive abilities are required for social behaviors. This theory, known as the social brain hypothesis, suggests that children naturally learn cooperation and competition (Bjorklund, 2018). In a study by Bakırcı et al. (2023), it is noted that “students forget where and how to speak and behave”. In fact, norms are the criteria of social life that can be learned through observation and experience throughout the life process.

At the end of this research, another noticeable behavior observed by teachers in children was found in the motor skills category. Specifically, noticeable behaviors such as “struggling with drawing, holding a pencil, and self-care skills” in preschool students, “struggling with writing” in primary school students, and “struggling with note-taking” in middle school students were observed. This situation can also be interpreted as indicating that actions requiring skills, learning, and activities were insufficient during the online education process. It is understood that students were not able to develop themselves as much in online education as they did in face-to-face education. Especially in skills that require practice, it can be said that students did not show sufficient development. Individuals who benefit from education to support all types of development did not benefit from online education as much as they did from face-to-face education. Even in areas where development could have been achieved at home with parental support, noticeable behaviors were observed in students. For example, many preschool students exhibited noticeable behaviors such as “struggling with self-care skills”, such as “washing hands, brushing teeth, personal hygiene, etc.” Additionally, another noticeable motor behavior observed in primary and middle school students was “struggling with writing/taking notes”. Due to the COVID-19 virus outbreak, it was found that students, especially young children in preschool and primary school ages, did not achieve the desired level of motor skill development. Therefore, actions such as drawing, painting, writing, making pictures, holding a racket, or playing a musical instrument are fine motor movements (Wang, 2004). It is clear that a process is required for the development of these skills. During online education, the conditions of school and class could not be provided at home.

The findings obtained in this study indicate that noticeable behaviors related to students’ affective skills were observed. For preschool students, noticeable behaviors such as “lack of interest in class”, “boredom during class”, “showing impatience”, and “difficulty expressing emotions” were observed. For elementary school students, “lack of interest in class” and “anxiety and worry” were noted. For middle school students, behaviors like “lack of interest in class”, “boredom during class”, and “aggression” were observed. For high school students, “lack of interest in class” and “aggression” were noticeable behaviors. The most noticeable teacher observations in the affective skills category for middle and high school students were “aggression and peer bullying”. The tendency for children who have been away from their friends for a long time to engage in such noticeable behaviors may be related to concepts like “identity formation” or “self-acceptance”. Another noticeable behavior observed at these educational levels was categorized as “lack of interest in class”. Emotions emerge in individuals from the earliest days of life (Sigelman & Rider, 2018) and describe what individuals feel toward events and people. Emotions are essential characteristics unique to the individual, playing an important role in regulating one’s own behavior and interacting with others (Santrock, 2014). It is clear that the COVID-19 pandemic, which threatens individuals’ lives, can create pessimistic thoughts, which may lead to anxiety and depression (Yüksel-Şahin & Öztoprak, 2019; Mochida et al., 2021; Li et al., 2020). Various research findings suggest that online education may reveal emotions such as boredom, worry, and anxiety in children (Yüksel-Şahin, 2021; Golberstein et al., 2020). Another noticeable behavior observed in middle and high school students in the affective skills category is “aggression/peer bullying”. The COVID-19 pandemic is considered a period that was full of uncertainties. Accordingly, during times of uncertainty, communication problems within families and social environments may lead to anger crises and behavioral problems (Koçak & Harmancı, 2020).

Another category defined in this study is social media addiction. Following the shift to online education during COVID-19, increased digital engagement and potential overuse of technology have been observed among students. At the preschool level, there is intense interest in visuals, while at the primary, middle, and high school levels, there is intense interest in social media. Additionally, at the high school level, there is also a habit of using slides observed. Children of this era are introduced to moving visuals from an early age, starting with the home environment. Parents sometimes give their children a tablet or a mobile phone to keep them occupied while they are busy with household chores. High school students, during their adolescent years, tend to intertwine both their education and leisure time with digital platforms, which naturally leads to their strong interest in social media (Akçay, 2020; Direktör, 2021). In addition to this, the increased necessity of the internet during the COVID-19 pandemic may have contributed to higher social media engagement and potential addiction (Derin & Bilge, 2016). Since all social media tools are dependent on the internet, this has led to the labelling of such behaviors as technology or social media addiction (Akçay, 2020; Anderson, 2020; Balcı et al., 2021). Moreover, while social media is perceived as an indispensable communication tool and a digital popularity mechanism, especially for adolescents, this technology also distracts individuals and hinders their focus, not only in their educational lives but also in their real social lives (Balcı et al., 2021; Hou et al., 2019; Üstün & Akın, 2022; Yüksel-Şahin & Öztoprak, 2019).

When the opinions of the study group are examined, it is observed that some of the noticeable student behaviors are also categorized under the personality characteristics category. These include egocentrism and adult dependency for preschool students; adult dependency and anger/aggression for elementary school students; selfishness and anger issues for middle school students; and insufficient personality development for high school students. It is clear that the anxiety and concerns caused by COVID-19 have negatively affected the psychology of children (See; Duan & Zhu, 2020; Dvorsky et al., 2020; Korukluoğlu & Bavlı, 2022; Orgilés et al., 2020; Pisano et al., 2020; Qiu et al., 2020; Saurabh & Ranjan, 2020; Shorer & Leibovich, 2020; Van Lancker & Parolin, 2020; Wendel et al., 2020). As a result, behaviors that normally would not be observed have become evident. According to teacher opinions, the noticeable behaviors coded under the personality traits category in elementary school children as anger and aggression, and in middle school children as anger issues, may be attributed to societal crisis situations. The self-centeredness observed in middle school students is considered to be one of the natural developmental characteristics of this age group (Piaget, 2005). Thus, the anger and aggressive behaviors observed in elementary and middle school students may be partially related to extended periods of home confinement and reduced social interaction during COVID-19. Children who need to move, explore, jump, play with their friends may not yet be able to rationalize the COVID-19 situation, or they may not have reached the cognitive development stage to understand its reality, which can lead them to exhibit angry and aggressive behaviors, since a child’s anger often stems from social reasons (Yavuzer et al., 2012). Moreover, the noticeable behavioral difference categorized as inadequate personality development was identified specifically among high school students within the personality traits category. To explain the expression coded as “inadequate personality development”, participants were asked to return interview forms via email, and when the responses were examined, it was understood that it referred to these student groups being far from the behaviors generally expected at their ages.

The rapidly spreading COVID-19 virus has profoundly affected societies worldwide. In Turkey, following the emergence of COVID-19 cases in March 2020, face-to-face education was suspended and online learning was adopted (Baykal, 2020; Can, 2020; Baz, 2021). However, children, who were confined to their homes and subjected to an almost mandatory social isolation, experienced neglect in their social, emotional, psychological, mental, behavioral, and academic development. For students, school is not merely a place to acquire knowledge: it is also a social environment where they interact with peers; discuss common goals, projects, and ideas; and learn to coexist, think critically, and recognize themselves as members of a community (Gutek, 2006; Schlechty, 2006). Attending school allows for children and young people to develop self-confidence and personal growth. Following the containment of COVID-19, students who had undergone online education returned to school for in-person learning. This transition was welcomed with joy, especially by children and their parents. However, many teachers, speaking in unison, have begun to express concerns regarding students’ behaviors upon their return to school. In this study, all of the noticeable behaviors observed by teachers in students after COVID-19 are prosocial behaviors that require in social learning environments. That is, if face-to-face education had not been interrupted due to COVID-19, children would likely not have forgotten these behaviors or could have learned what they did not know through social learning. According to Skinner, an individual first observes the behavior of a model, and then transforms their own behavior to match the observed model’s behavior. J. Dewey argues that “people can learn from the environment they live in and from the people they communicate with” (Özer & Hotaman, 2021). Tolman also views learning as a “process of exploring the environment” (Senemoğlu, 2020).

In conclusion, all teachers who participated in this study reported that after the online education implemented due to COVID-19, they experienced discipline problems regarding rule compliance, difficulties in focusing their cognitive attention, struggles in social communication, lack of interest in lessons emotionally, and a significant interest in social media, which could be considered as digital addiction, in preschool, primary school, middle school, and high school students who returned to school and face-to-face education.

## 6. Suggestions

In light of the findings from post-COVID-19 studies, it is considered beneficial to integrate the online Psychological Counseling and Guidance (PDR) services—initially offered by the Ministry of National Education during the pandemic—into the regular school-based PDR services to support students more effectively. Moreover, for students participating in online education as part of adaptation programs, it would be advantageous to remind them of school and classroom rules, treating them as if they are newly introduced to the school environment. Additionally, to address the frequent learning gaps observed among students, it is recommended that ongoing monitoring tests be conducted and followed by formative and remedial assessments at each unit level. These assessments should be designed both horizontally and vertically, establishing connections within and across subject units. Furthermore, for students exhibiting signs of disinterest and boredom during lessons, implementing student-centered, contemporary teaching methods—while considering the physical structure of classrooms and class sizes—may enhance engagement. Where possible, the use of out-of-school learning environments should be expanded through careful planning and coordination to replace more routine class or school-based activities. Given the evident need for students to socialize and communicate with peers, it is also suggested that educators incorporate group activities and further integrate rarely utilized out-of-school settings into the curriculum to help repair weakened group dynamics and foster educational collaboration. In addition to maintaining the Ministry’s PDR services, offering psychiatric support tailored to students’ mental and emotional conditions is essential for strengthening individual counselling and facilitating access to those in need. Simultaneously, there is a pressing need to revisit not only curricular content but also school environments in accordance with innovative educational approaches that promote social interaction among students. To improve observed behavioral challenges related to norms, general adaptation, and social life rules, the development of affective education-based teaching programs is recommended. Finally, the Ministry of National Education, along with schools and other stakeholders, should systematically monitor the long-term effects of the negative patterns identified in such studies. This will be vital for safeguarding student well-being and for enhancing the overall human quality of the Turkish education system, while also identifying potential solutions for these emerging concerns.

## Figures and Tables

**Table 1:** Data regarding the study group.

School Level	Preschool Seniority	Primary School Seniority	Middle School Seniority	High School Seniority	Total Seniority
Total	22 05–20	26 06–30	25 10–35	23 10–30	96 05–35

**Table 2:** Content analysis results of data obtained from preschool teachers.

Theme	Category	Code	f	Participants
Noticeable behaviors (preschool)	Discipline	Difficulty following the rules	12	1, 2, 7, 8, 9, 12, 13, 15, 16, 17, 18, 20
	Cognitive Skills	Difficulty taking directions	13	4, 5, 6, 7, 9, 11, 12, 13, 15, 16, 18, 20, 21
		Difficulty in problem solving	08	4, 5, 7, 11, 16, 17, 18, 20
		Difficulty concentrating	16	1, 2, 3, 4, 5, 6, 7, 9, 10, 12, 13, 15, 16, 17, 19, 21
	Social Skills	Difficulty making friends	12	4, 5, 6, 9, 11, 13, 14, 16, 17, 18, 20, 21
		Difficulty communicating	11	1, 4, 5, 6, 8, 12, 16, 17, 19,20, 21
	Motor Skills	Difficulty drawing	14	1, 2, 3, 4, 5, 6, 7, 8, 11, 13, 15, 17, 19, 21
		Difficulty holding a pencil	11	1, 2, 4, 5, 6, 8, 11, 14, 16, 17, 19
		Difficulty in self-care skills	11	1, 3, 4, 5, 6, 8, 10, 14, 16, 17, 20
	Affective Skills	Indifference to the lesson	15	1, 2, 4, 5, 6, 7, 8, 9, 13, 14, 16, 17, 18, 19, 22
		Bored in class	10	1, 3, 5, 6, 9, 10, 13, 16, 18, 19
		Showing impatience	06	2, 5, 7, 19, 20, 21
		Difficulty expressing emotions	05	6, 12, 14, 17, 19
	Digital Addiction	Intense interest in visuals	15	1, 2, 4, 5, 6, 7, 8, 9, 13, 14, 16, 17, 18, 19, 22
	Personality characteristics	Self-centeredness	05	9, 12, 17, 18, 20
		Adult dependence	05	3, 7, 10, 19, 20

**Table 3:** Content analysis results of data obtained from primary school teachers.

Theme	Category	Code	f	Participants
Noticeable behavioral differences (primary school)	Discipline	Difficulty following the rules	12	2, 3, 5, 8, 9, 11, 12, 14, 16, 17, 22, 23
		Difficulty conforming to norms	09	1, 4, 12, 14, 16, 19, 20, 22, 23
	Cognitive skills	Difficulty in learning	23	1, 2, 3, 4, 5, 7, 8, 9, 10, 11, 12, 13, 15, 16, 17, 18, 19, 20, 21, 22, 23, 25, 26
		Difficulty concentrating	13	5, 9, 11, 12, 13, 16, 18, 19, 20, 22, 23, 24, 26
	Social skills	Difficulty making friends	06	2, 7, 9, 13, 17, 20
		Difficulty communicating	06	2, 7, 9, 13, 16, 17
		Difficulty in getting along with peers	06	2, 7, 9, 13, 16, 19
	Motor skills	Difficulty in writing	09	2, 5, 9, 10, 12, 17, 19, 21, 24
	Affective skills	Indifference to the lesson	07	1, 2, 5, 7, 12, 15, 16
		Anxiety/worry	05	1, 2, 7, 14, 25
	Digital addiction	Intense interest in social media	05	1, 7, 11, 12, 18
	Personality characteristics	Adult dependence	05	12, 16, 17, 19, 20
		Anger/aggression	06	4, 9, 12, 16, 19, 22, 23

**Table 4:** Content analysis results of data obtained from middle school teachers.

Theme	Category	Code	f	Participants
Noticeable behavioral differences (middle school)	Discipline	Difficulty following the rules	15	1, 2, 3, 4, 5, 6, 7, 10, 12, 13, 14, 17, 18, 23, 24
		Difficulty conforming to norms	07	3, 5, 6, 7, 10, 18, 24
	Cognitive skills	Difficulty in learning	06	1, 8, 9, 11, 21, 25
		Difficulty concentrating	13	1, 4, 5, 7, 8, 9, 11, 15, 17, 20, 22, 24, 25
		Difficulty in reading	12	1, 3, 8, 9, 13, 16, 17, 18, 21, 22, 23, 25
	Social skills	Difficulty making friends	09	2, 3, 5, 6, 13, 20, 21, 24, 25
		Difficulty communicating	08	1, 2, 4, 5, 6, 9, 11, 19
	Motor skills	Difficulty taking/writing notes	05	3, 11, 12, 13, 20
	Affective skills	Indifference to the lesson	11	1, 4, 5, 8, 9, 10, 13, 15, 18, 24, 25
		Bored in class	08	5, 8, 9, 10, 13, 15, 24, 25
		Aggression/peer bullying	12	1, 4, 5, 6, 7, 11, 12, 13, 15, 16, 17, 19
	Digital addiction	Intense interest in social media	08	1, 5, 7, 9, 13, 17, 21, 25
	Personality characteristics	Self-centeredness	07	2, 3, 4, 6, 15, 17, 18
		Anger problem	06	1, 4, 5, 17, 19, 22

**Table 5:** Content analysis results of data obtained from high school teachers.

Theme	Category	Code	F	Participants
Noticeable behavioral differences (high school)	Discipline	Difficulty following the rules	10	1, 3, 4, 6, 7, 11, 13, 16, 19, 20, 21
		Difficulty conforming to norms	11	3, 5, 6, 7, 14, 15, 16, 20, 21, 22, 23
		Slang/cursing habit	06	2, 3, 6, 12, 19, 22
	Cognitive skills	Difficulty concentrating	07	3, 5, 8, 12, 14, 15, 17
		Lack of learning	15	1, 2, 3, 5, 6, 7, 10, 11, 14, 15, 17, 19, 20, 21, 22
	Social skills	Difficulty communicating	13	1, 2, 3, 5, 6, 8, 11, 12, 13, 19, 20, 22, 23
	Affective skills	Indifference to the lesson	16	1, 2, 3, 4, 6, 7, 8, 9, 11, 12, 15, 16, 17, 21, 22, 23
		Aggression/peer bullying	09	2, 3, 5, 6, 7, 12, 13, 21, 22
	Digital addiction	Intense interest in social media	13	1, 3, 5, 7, 9, 12, 14, 17, 19, 20, 21, 22, 23
		Habit of relying on the slides	05	9, 13, 15, 17, 23
	Personality characteristics	Inadequate personality development	07	8, 13, 14, 15, 17, 19, 20

## Data Availability

The datasets generated and analyzed during this study are available from the author on reasonable request.

## References

[B1-behavsci-15-00554] Akçay D. (2020). Ebeveynlerin çocukların dijital video oyunları bağımlılığı karşısında tutum ve davranışları [Parents’ attitudes and behaviors towards children’s digital video game addiction]. Middle Black Sea Journal of Communication Studies.

[B2-behavsci-15-00554] Anderson J. (2020). Brave new world the coronavirus pandemic is reshaping education.

[B3-behavsci-15-00554] Bacanlı H. (2021). Eğitim psikolojisi [Educational psychology].

[B4-behavsci-15-00554] Bakırcı H., Urhan B., Bülbül S., İlhan R. (2023). Teachers’ opinions on students’ adaptation to school after the COVID-19 pandemic. International e-Journal of Educational Studies.

[B5-behavsci-15-00554] Balcı E., Durmuş H., Sezer L. (2021). Corona günlerinde uzaktan eğitim bağımlılık gelişiminde bir risk oluşturur mu? [Does remote education during the days of Corona pose a risk for addiction development?]. Bağımlılık Dergisi.

[B6-behavsci-15-00554] Baykal E. (2020). COVID-19 bağlaminda psikolojik dayaniklilik, kaygi ve yaşam doyum ilişkisi [The relationship between psychological resilience, anxiety, and life satisfaction in the context of COVID-19]. International Journal of Social and Economic Sciences.

[B7-behavsci-15-00554] Baz B. (2021). COVID-19 salgini sürecinde öğrencilerin olasi öğrenme kayiplari üzerine bir değerlendirme [An assessment on possible learning losses in students during the COVID-19 pandemic]. Temel Eğitim Dergisi.

[B8-behavsci-15-00554] Bilen M. (2009). Sağlıklı insan ilişkileri [Healthy human relationships].

[B9-behavsci-15-00554] Bjorklund D. F. (2018). A metatheory for cognitive develoment (Or “piaget is dead” revisited). Child Development.

[B10-behavsci-15-00554] Bozkurt V. (2006). Değişen dünyada sosyoloji: Temeller, kavramlar, kurumlar [Sociology in a changing world: Foundations, concepts, institutions].

[B11-behavsci-15-00554] Brym R. J. (1998). New society: Sociologyfor 21st century.

[B12-behavsci-15-00554] Can E. (2020). Coronavirus (COVID-19) pandemisi ve pedagojik yansımaları: Türkiye’de açık ve uzaktan eğitim uygulamaları [Coronavirus (COVID-19) pandemic and its pedagogical implications: Open and distance education practices in Turkey]. Açıköğretim Uygulamaları ve Araştırmaları Dergisi.

[B13-behavsci-15-00554] Creswell J. W. (2017). Research design: Qualitative, quantitative, and mixed methods approaches.

[B14-behavsci-15-00554] Çubukçu Z., Gültekin M. (2006). İlköğretimde öğrencilere kazandırılması gereken sosyal beceriler [The social skills to be acquired by primary school students]. Bilig.

[B15-behavsci-15-00554] Demirel Ö. (2021). Öğretim ilke ve yöntemleri: Öğretme sanati [Principles and methods of instruction: The art of teaching].

[B16-behavsci-15-00554] Derin S., Bilge E. (2016). Ergenlerde internet bağimliliği ve öznel iyi oluş düzey [Internet addiction and subjective well-being in adolescents]. Türk Psikolojik Danışma ve Rehberlik Dergisi.

[B17-behavsci-15-00554] Direktör C. (2021). COVID-19 salgini ve çocuk psikolojisi [COVID-19 pandemic and child psychology]. Psikiyatride Güncel Yaklaşımlar Dergisi.

[B18-behavsci-15-00554] Duan L., Zhu G. (2020). Psychological interventions for people affected by the COVID-19 epidemic. The Lancet. Psychiatry.

[B19-behavsci-15-00554] Dvorsky M. R., Breaux R., Becker S. P. (2020). Finding ordinary magic in extraordinary times: Child and adolescent resilience during the COVID-19 pandemic. European Child & Adolescent Psychiatry.

[B20-behavsci-15-00554] Falowo R. O. (2007). Factors impeding implementation of web-based distance learning. AACE Journal.

[B21-behavsci-15-00554] Golberstein E., Wen H., Miller B. F. (2020). Coronavirus disease 2019 (COVID-19) and mental health for children and adolescents. JAMA Pediatrics.

[B22-behavsci-15-00554] Guba E. G., Lincoln Y. S., Denzin N. K., Lincoln Y. S. (2005). Paradigmatic controversies, contradictions, and emerging confluences. The Sage handbook of qualitative research.

[B23-behavsci-15-00554] Gutek G. L., Kale N. (2006). Philosophical and ideological perspectives on education.

[B24-behavsci-15-00554] Hennink M., Kaiser B. N. (2022). Sample sizes for saturation in qualitative research: A systematic review of empirical tests. Social Science & Medicine.

[B25-behavsci-15-00554] Hotaman D., Yapıcı M. (2018a). Davranışın doğası [Nature of behavior]. Psychological processes from birth to death: Psychology.

[B26-behavsci-15-00554] Hotaman D. (2018b). Toplumsal ve evrensel ihtiyaçlara göre okulun işgörüsünü yeniden tanımlamak [Redefining the school’s mission according to societal and universal needs]. YILDIZ Journal of Educational Research.

[B27-behavsci-15-00554] Hou Y., Xiong D., Jiang T., Song L., Wang Q. (2019). Social media addiction: Its impact, mediation, and intervention. Cyberpsychology: Journal of Psychosocial Research on Cyberspace.

[B28-behavsci-15-00554] Kaffenberger M. (2021). Modelling the long-run learning impact of the COVID-19 learning shock: Actions to (more than) mitigate loss. International Journal of Educational Development.

[B29-behavsci-15-00554] Koçak Z., Harmancı H. (2020). COVID-19 pandemi sürecinde ailede ruh sağlığı [Family mental health during the COVID-19 pandemic]. Karatay Sosyal Araştırmalar Dergisi.

[B30-behavsci-15-00554] Korukluoğlu P., Bavlı B. (2022). Öğrenci velileri gözünden COVID-19 Pandemisi acil uzaktan öğretim (AUÖ) süreci: Fenomenolojik bir çalışma [The COVID-19 pandemic emergency remote learning (ERL) process from the perspective of student parents: A phenomenological study]. Afyon Kocatepe Üniversitesi Sosyal Bilimler Dergisi.

[B31-behavsci-15-00554] Kuhfeld M., Tarasawa B., Johnson A., Ruzek E., Lewis K. (2020). Learning during COVID-19: Initial findings on students’ reading and math achievement and growth.

[B32-behavsci-15-00554] Li H., Liu S. M., Yu X. H., Tang S. L., Tang C. K. (2020). Coronavirus disease 2019 (COVID-19): Current status and future perspectives. International Journal of Antimicrobial Agents.

[B33-behavsci-15-00554] Merriam S. B. (2009). Qualitative research. A guide to design and implementation.

[B34-behavsci-15-00554] Merriam S. B., Grenier R. S. (2019). Qualitative research in practice: Examples for discussion and analysis.

[B35-behavsci-15-00554] Merriam S. B., Tisdell E. J. (2015). Qualitative research: A Guide to design and implementation.

[B36-behavsci-15-00554] Miles M. B., Huberman A. M. (1994). Qualitative data analysis: A sourcebook.

[B37-behavsci-15-00554] Mochida S., Sanada M., Shao Q., Lee J., Takaoka J., Ando S. (2021). Factors modifying children’s stress during the COVID-19 pandemic in Japan. European Early Childhood Education Research Journal.

[B38-behavsci-15-00554] Moore M. G., Kearsley G. (2011). Distance education: A systems view of online learning.

[B39-behavsci-15-00554] Mutlu H., Güner B., Doğanay G., Yılmaz D. (2020). Veli algılarına göre pandemi dönemi uzaktan eğitim sürecinin niteliği [The quality of the pandemic period distance education process according to parent perceptions] *(Research Coordinator: Ercan Yılmaz). Palet Yayınları*.

[B40-behavsci-15-00554] Orgilés M., Morales A., Delvecchio E., Mazzeschi C., Espada J. P. (2020). Immediate psychological effects of the COVID-19 quarantine in youth from Italy and Spain. Frontiers in Psychology.

[B41-behavsci-15-00554] Özden Y. (2000). Öğrenme ve öğretme [Learning and teaching].

[B42-behavsci-15-00554] Özer E., Hotaman D., Hotaman D. (2021). Bilişsel davranişçilar [Cognitive behaviorists]. Learning psychology.

[B43-behavsci-15-00554] Patton M. Q., Demir S. B., Bütün M. (2018). Qualitative research and evaluation methods.

[B44-behavsci-15-00554] Piaget J., Gabain M. (2005). The child’s conception of the world.

[B45-behavsci-15-00554] Pisano L., Galimi D., Cerniglia L. (2020). A qualitative report on exploratory data on the possible emotional/behavioral correlates of COVID-19 lockdown in 4–10 years children in Italy. PsyArxiv.

[B46-behavsci-15-00554] Qiu J., Shen B., Zhao M., Wong Z., Xie B., Xu Y. (2020). A nationwide survey of psychological distress among Chinese people in the COVID-19 epidemic: Implications and policy recommendations. General Psychiatry.

[B47-behavsci-15-00554] Santrock J. W. (2014). Child development.

[B48-behavsci-15-00554] Saurabh K., Ranjan S. (2020). Compliance and psychological impact of quarantine in children and adolescents due to COVID19 pandemic. Indian Journal of Pediatrics.

[B49-behavsci-15-00554] Schlechty P. C., Yüksel Ö. (2006). Okulu yeniden kurmak (Shaking up the school house: How to support and sustain educational innovation).

[B50-behavsci-15-00554] Senemoğlu N. (2020). Gelişim, öğrenme ve öğretme: Kuramdan uygulamaya.

[B51-behavsci-15-00554] Shorer M., Leibovich L. (2020). Young children’s emotional stress reactions during the COVID-19 outbreak and their associations with parental emotion regulation and parental playfulness. Early Child Development and Care.

[B52-behavsci-15-00554] Sigelman C. K., Rider E. A. (2018). Life-span human development.

[B53-behavsci-15-00554] Sönmez V. (2009). Öğretim ilke ve yöntemleri.

[B54-behavsci-15-00554] Strauss A., Corbin J. (1990). Basics of qualitative research: Grounded theory procedures and techniques.

[B55-behavsci-15-00554] Streubert H. J., Carpenter D. R. (2011). Qualitative research in nursing.

[B56-behavsci-15-00554] Til A. (2020). Yeni koronavirüs hastalığı hakkında bilinmesi gerekenler. Ayrıntı Dergisi.

[B57-behavsci-15-00554] Üstün A., Akın E. (2022). Özel öğretim kurumlarindaki öğretmen görüşlerine göre lise öğrencilerindeki teknoloji bağimliliği [Technology addiction in high school students according to teacher opinions in private educational institutions]. International Academic Social Resources Journal.

[B58-behavsci-15-00554] Van Lancker W., Parolin Z. (2020). COVID-19, school closures, and child poverty: A social crisis in the making. The Lancet Public Health.

[B59-behavsci-15-00554] Wang J. H. (2004). A study on gross motor skills of preschool children. Journal of Research in Childhood Education.

[B60-behavsci-15-00554] Weber M. B. (2019). What influences saturation? Estimating sample sizes in focus group research. Qualitative Health Research.

[B61-behavsci-15-00554] Wendel M., Ritchie T., Rogers M. A., Ogg J. A., Santuzzi A. M., Shelleby E. C., Menter K. (2020). The association between child ADHD symptoms and changes in parental involvement in kindergarten children’s learning during COVID-19. School Psychology Review.

[B62-behavsci-15-00554] Wentzel K. R. (2003). Motivating students to behave in socially competent ways. Theory Into Practice.

[B63-behavsci-15-00554] WHO (2020). WHO Director-General’s opening remarks at the media briefing on 2019 novel coronavirus.

[B64-behavsci-15-00554] Wilson A. (2015). A guide to phenomenological research. Nursing Standard.

[B65-behavsci-15-00554] Yavuzer Y., Demir Z., Çalışkan M. (2012). Eğitim psikolojisi: Gelişim ve öğrenme [Educational psychology: Development and learning].

[B66-behavsci-15-00554] Yavuzer Y., Üre Ö. (2010). Saldırganlığı Önlemeye Yönelik Psiko-Eğitim Programlarının Lise Öğrencilerindeki Saldırganlığı Azaltmaya Etkisi. Selçuk Üniversitesi Sosyal Bilimler Enstitüsü Dergisi.

[B67-behavsci-15-00554] Yin R. (2014). Case study research: Design and methods.

[B68-behavsci-15-00554] Yıldırım A., Şimşek H. (2018). Sosyal bilimlerde nitel araştırma yöntemleri [Qualitative research methods in social sciences].

[B69-behavsci-15-00554] Yıldız S., Bektaş F. (2020). COVID-19 salgininin çocuklarin boş zaman etkinliklerinde yarattiği değişimin ebeveyn görüşleriyle değerlendirilmesi [Evaluation of the impact of the COVID-19 pandemic on children’s leisure activities through parental perspectives]. Gazi Journal of Physical Education and Sport Sciences.

[B70-behavsci-15-00554] Yüksel-Şahin F. (1997). Grupla iletişim becerileri eğitiminin üniversite öğrencilerinin iletişim beceri düzeylerine etkisi [The impact of group communication skills training on university students’ communication skill levels. Ph.D. dissertation.

[B71-behavsci-15-00554] Yüksel-Şahin F., Kaya A. (2020). İletişim becerilerine genel bir bakış [An overview of communication skills]. Human relations and communication.

[B72-behavsci-15-00554] Yüksel-Şahin F. (2021). Psikolojik danışmanların Covid-19’a, yüz yüze ve çevrimiçi psikolojik danışma yapmaya, yüz yüze ve çevrimiçi eğitim almaya ilişkin görüşlerinin incelenmesi. IBAD Sosyal Bilimler Dergisi.

[B73-behavsci-15-00554] Yüksel-Şahin F., Öztoprak Ö. (2019). Ergenlerin sosyal medya bağimliliği düzeylerinin benlik saygisina göre incelenmesi [Examination of adolescents’ social media addiction levels according to self-esteem] (Special ed.). IBAD-Sosyal Bilimler Dergisi.

